# Role of T1 mapping as a complementary tool to T2* for non-invasive cardiac iron overload assessment

**DOI:** 10.1371/journal.pone.0192890

**Published:** 2018-02-21

**Authors:** Camilla Torlasco, Elena Cassinerio, Alberto Roghi, Andrea Faini, Marco Capecchi, Amna Abdel-Gadir, Cristina Giannattasio, Gianfranco Parati, James C. Moon, Maria D. Cappellini, Patrizia Pedrotti

**Affiliations:** 1 Department of Cardiovascular, Neural and Metabolic Sciences, San Luca Hospital, Istituto Auxologico Italiano, Milan, Italy; 2 Rare Diseases Centre, Department of Medicine and Medical Specialities, “Ca’ Granda” Foundation IRCCS, Milan, Italy; 3 Cardiology 4, Department of Cardiology and Cardiovascular Surgery, Niguarda Hospital, Milan, Italy; 4 Institute of Cardiovascular Science, University College London, London, United Kingdom; Universitatsklinikum Wurzburg, GERMANY

## Abstract

**Background:**

Iron overload-related heart failure is the principal cause of death in transfusion dependent patients, including those with Thalassemia Major. Linking cardiac siderosis measured by T2* to therapy improves outcomes. T1 mapping can also measure iron; preliminary data suggests it may have higher sensitivity for iron, particularly for early overload (the conventional cut-point for no iron by T2* is 20ms, but this is believed insensitive). We compared T1 mapping to T2* in cardiac iron overload.

**Methods:**

In a prospectively large single centre study of 138 Thalassemia Major patients and 32 healthy controls, we compared T1 mapping to dark blood and bright blood T2* acquired at 1.5T. Linear regression analysis was used to assess the association of T2* and T1. A “moving window” approach was taken to understand the strength of the association at different levels of iron overload.

**Results:**

The relationship between T2* (here dark blood) and T1 is described by a log-log linear regression, which can be split in three different slopes: 1) T2* low, <20ms, r2 = 0.92; 2) T2* = 20-30ms, r2 = 0.48; 3) T2*>30ms, weak relationship. All subjects with T2*<20ms had low T1; among those with T2*>20ms, 38% had low T1 with most of the subjects in the T2* range 20-30ms having a low T1.

**Conclusions:**

In established cardiac iron overload, T1 and T2* are concordant. However, in the 20-30ms T2* range, T1 mapping appears to detect iron. These data support previous suggestions that T1 detects missed iron in 1 out of 3 subjects with normal T2*, and that T1 mapping is complementary to T2*. The clinical significance of a low T1 with normal T2* should be further investigated.

## Introduction

Iron overload-related heart failure is the principal cause of death in transfused Thalassemia Major (TM) and other iron overload patients. [1,2] Iron toxicity is dose dependent so a strategy of chelation therapy titration against measured iron dose [3,4] before the onset of left ventricle (LV) impairment improves outcomes. [5] The presence of iron in tissues determines microscopic magnetic field inhomogeneity and thus changes the magnetic properties of water, T1, T2 and T2*. This has been validated against tissue in animal and human models. [6,7,8] To date, T2* has been the most used technique to non-invasively assess cardiac iron load. T2* would be normal above 40ms ([Fe] = 0.50mg/g dw; ex-vivo calibration) [9] but in practice, a threshold of 20ms ([Fe] = 1.1mg/g dw, 2x normal) is used to avoid false positives. A higher threshold would reduce accuracy, because T2* at low iron levels has sensitivity to non-iron influences (susceptibility artefact). This is reflected by the exponential T2* standard deviation (SD) relationship with increasing T2*, meaning an increased inter-operator variability for T2*>20ms. [10] These technical limitations persist in spite of the development of a dark blood T2* sequence (DBT2*), which has been demonstrated to be more reproducible and accurate than the older bright blood T2* (BBT2*). [11] With DBT2*, a double inversion recovery pulse is used to null the signal from blood and the multi-echo T2* images are acquired in late diastole, when cardiac motion is negligible. [12] Also, T2* used to require post processing, but a new free-breathing T2* mapping using respiratory motion corrected averaging has recently been developed. [13,14]

T1mapping may help. [15–17] It correlates with cardiac and liver iron in animal models, better than T2* for high iron load. [6] In humans, data from Pennell et al shows that ex-vivo T1 correlates with cardiac iron (hearts fixed with formalin for <10weeks), while in-vivo it is used for measuring intramyocardial haemorrhage. [18] Moreover, T1 mapping works at 3T [19], is less susceptible to artefacts [15,16] and mapping means post-processing is not required. [16,17] To date, few papers have explored T1 mapping in cardiac siderosis in-vivo, [19, 20, 21] both demonstrating superior inter-study reproducibility (2.5-7times) and T1 reclassification of a significant proportion of patients as having mild iron overload. However, sample size was small and the curve fit was unclear between T1 and T2*. Accordingly, we prospectively explored T1 and T2* with state-of-the-art sequences in a large cohort of TM patients seeking to understand the relationship of T1 and T2* in vivo and the potential clinical utility of combined T1 and T2* mapping.

## Methods

All participants provided written informed consent for the study, approved by IRCCS Fondazione Policlinico CA' Granda Ethics Committee. The study was conducted accordingly to the 1975 Helsinki Declaration.

### Populations

Cardiac magnetic resonance (CMR) scans of 140 TM patients studied between July 2013 and February 2016 were analysed, along with matching CMR scans of 32 healthy volunteers (HV), i.e. subjects with normal CMR scan and without known chronic medical conditions or pharmacological therapy.

### Cardiac magnetic resonance

Long- and short-axis, LV SSFP cine images, LV BBT2*, DBT2* and T1 mapping sequences (modified look locker inversion recovery–MOLLI–Siemens Works in Progress 448B) were acquired on an Avanto 1.5T scanner (Siemens Healthcare, Erlangen, Germany). For both DB and BB T2* sequences, the same single 10mm mid-ventricular short axis slice was imaged using a single breath-hold ECG-gated gradient multi-echo technique to generate eight images with a range of echo times (1.56ms to 17.17ms, increment 2.23ms), flip angle = 20°, FOV read/phase = 400mm/68.8%. The same slice was used for T1 images (thickness = 6mm, distance factor = 67%, FOV read/phase = 360/75%, TR = 740, TE = 1.13, 5+2 with motion correction for the in-line map generation). Quality control of T1 maps was performed by review of the error maps generated with the sequence and review of the raw images.

Two experienced operators independently analysed the scans. A single region of interest was manually traced in the interventricular septum avoiding the endo- and epicardial contours on the T1 maps. T2* measurements were carried out using the CMRTools “Thalassemia-Tools” plug-in (Cardiovascular Imaging Solutions, London, UK). Signal intensity was plotted against each echo time and the T2* value was calculated from the resulting exponential decay curve. Truncation method was used to account for the plateau observed at the later echo times due to signal loss. [22] (S1 Fig).

### Clinical data

For each patient demographical and anthropometric information, transfusion regimen, chelation therapy, pre-transfusion haemoglobin and ferritin were collected.

### Statistical analysis

Descriptive statistics were expressed as mean±SD for continuous variables and as frequency distribution for discrete variables. To assess the association between DBT2* and either BBT2* and T1, a linear regression analysis was performed in a log-log scale. To detect where DBT2* and T1 association decreases, the determination coefficient was computed in a log-log scale with 30 samples of moving window over DTB2* with steps of 1 sample at a time. To smooth such series a low-pass filter based on moving average of order 3 was applied. Afterward, a new equispaced series was generated by linear interpolation. The linear regression fall was identified visually as the local maximum immediately before the large variation of the determination coefficient and the numerical value corresponding to that point was found by setting the first derivative equal to 0. Considering the methodology limits (oscillation of the determination coefficient r2 by 10% and visual inspection method), which make impossible the definition of a sharp cut-off point, the numerical value obtained was rounded and used to identify an T2* values interval.

An alpha level of 0.05 was used. All analyses were performed using R Core Team (2015, Vienna, Austria).

## Results

All subjects underwent CMR without complications. Two patients and no HVs were excluded due to artefact in more than one sequence: DBT2* n = 3 (2%); BBT2* n = 4 (3%) and T1 n = 1 (<1%). Table 1 illustrates patients’ and HV’s characteristics. Raw data available at S1 File.

**Table 1:** Baseline patient characteristics.

	Patients (n = 138)	Healthy volunteers (n = 32)
**Age (years)**	37.2±4	38.1±15
**Gender (M:F)**	60:79	27:5
**BSA**	1.6±0.1	1.9±0.1
**Ferritin (g/dl)**	720 (170–5943)	-
**Hb (g/L)**	9.7 (8.3–11.6)	-

Normal data are expressed as mean±SD, non-normal data are expressed as median (2.5–97.5 percentiles). *BSA*: Body Surface Area. *Hb*: Haemoglobin.

**Fig 1:**
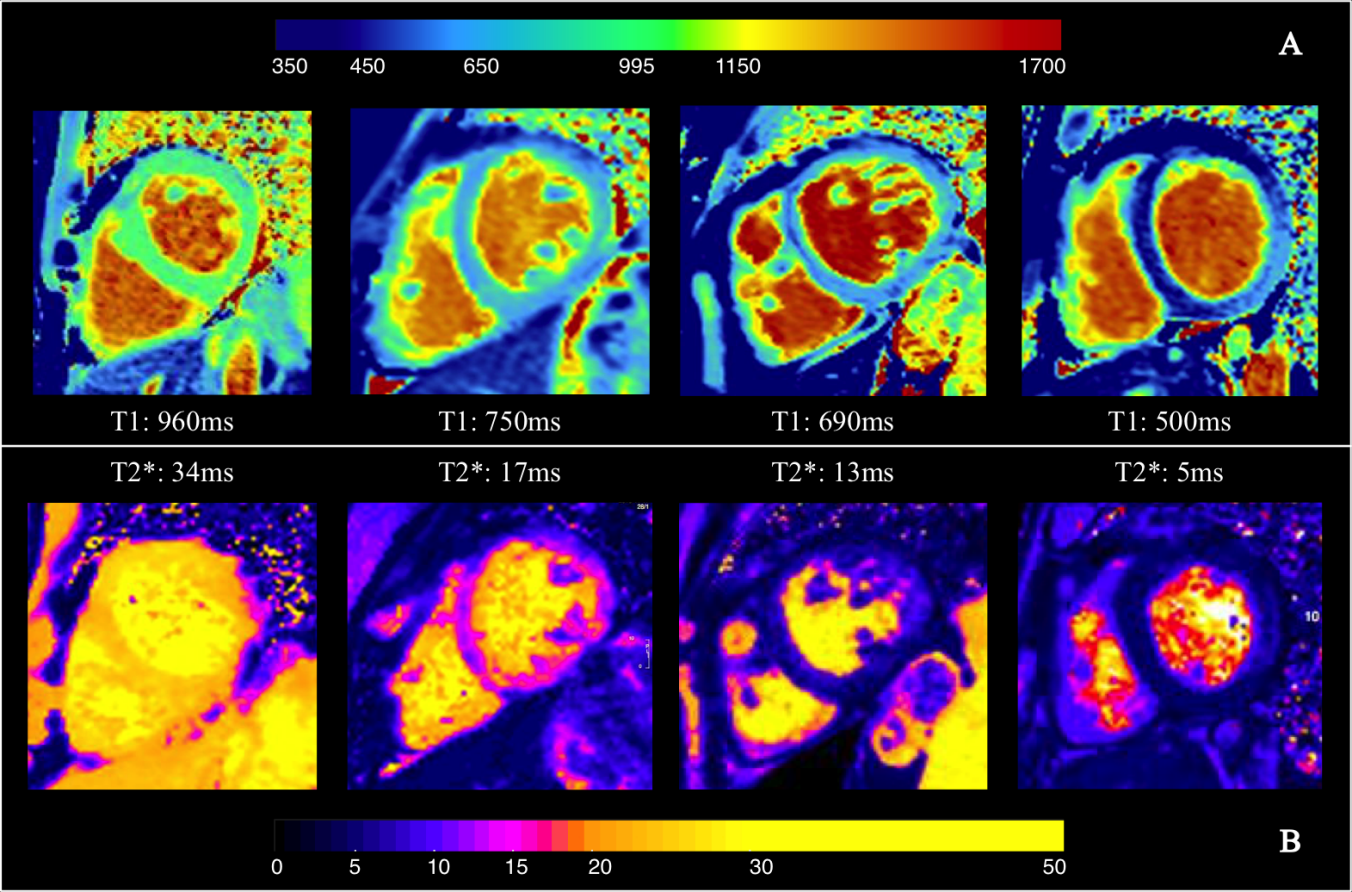
Examples of paired T1 mapping and corresponding T2* map. Panel A: T1 mapping, with colour scale bar on top. Panel B: T2* map, with colour scale bar below. In both panels, from left to right, data from a normal volunteer, a patient with mild iron overload, a patient with moderate iron overload and a patient with severe iron overload, are separately shown. Colour scale for T1 map is on top, for T2* map is at the bottom of the picture. Low T1, corresponding to iron, is represented in blue in the T1 maps, and low T2* is represented in pink-to-blue.

In the patients’ cohort median DBT2* was 34.3ms (full range 3.8–52.8ms), median BBT2* 35.9ms (full range 4.2–77.7ms) and median T1 926ms (2.5–97.5 percentiles 594-1003ms, full range 452-1062ms). Normality for T2* was defined by the internationally accepted 20ms cut-point. The T1 normal range was defined by the HV cohort as 918-1015ms (the 2.5–97.5 percentiles with CI 95%, as values were not normally distributed). Full range was 913-1050m. Fig 1.

**Fig 2:**
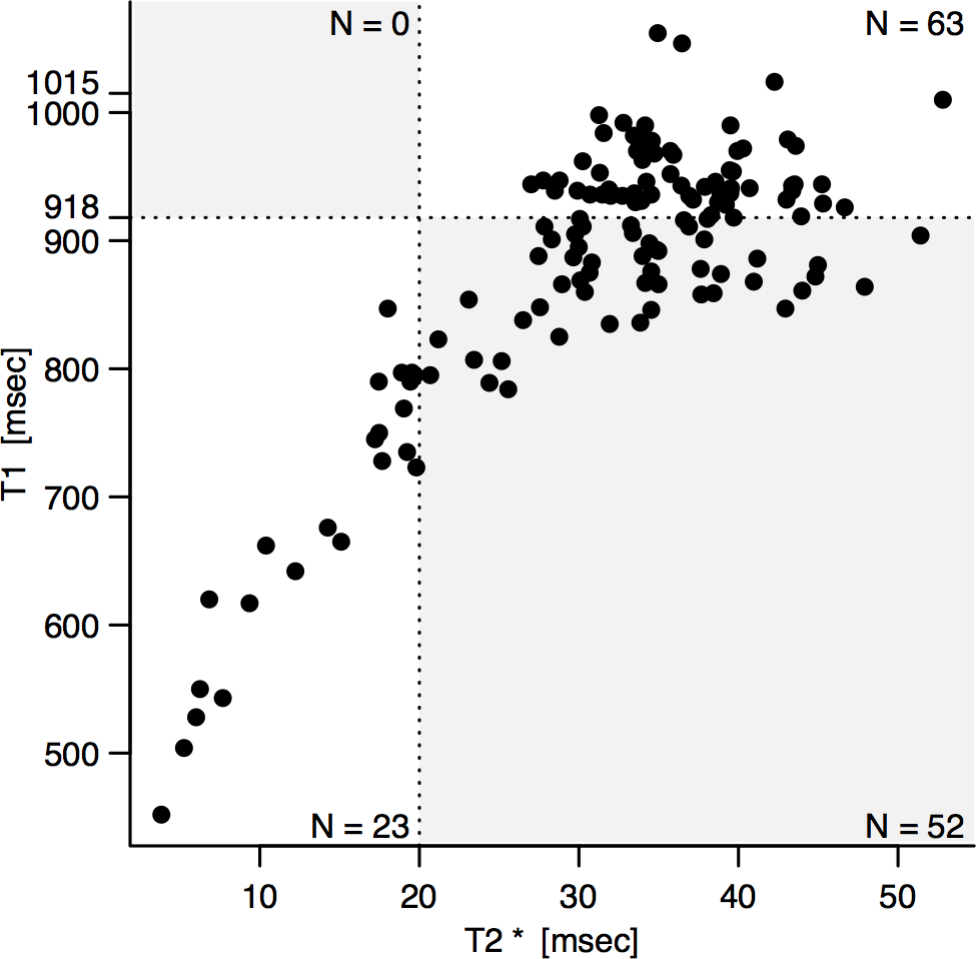
T2* vs T1 in Thalassemia major patients. The dashed lines highlight the cut off points for T2* (here Dark Blood, conventionally 20ms) and T1 (MOLLI, 916ms, as calculated from the healthy volunteer cohort). Dots localised in the grey quadrants represents discordance. All subjects with a discordant classification (n = 62, 38% of the cohort) fall into the lower right panel, i.e. they are characterized by normal T2*(iron not present), low T1 (iron present). Thus, apparently, T2* fails to identify 2 out of every 3 patients with cardiac iron overload.

**Fig 3:**
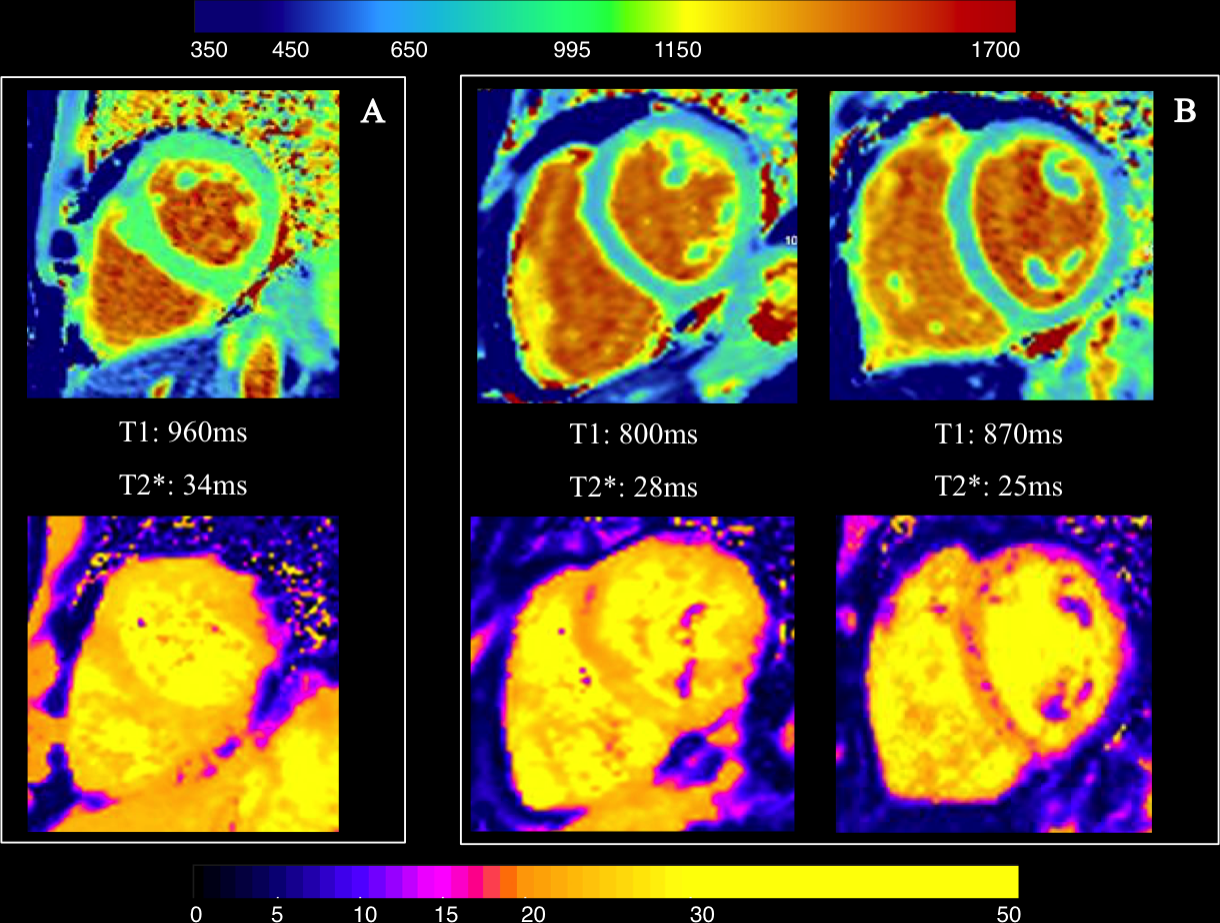
Examples of paired T2*-T1 non-concordance. Panel A: T2* and T1 map in a healthy volunteer. Panel B: T1 map and T2* map of two patients with normal T2* but low T1. Colour scale for T1 map is on top, for T2* map is at the bottom of the picture. Please note the blue shift of the myocardium in the T1 maps represented in panel B, compared to the healthy volunteer. Similarly, some pink areas may be spotted in the corresponding T2* maps, absent in the healthy volunteer. Nevertheless, dark blood T2* analysis performed with CMR Tools Thalassemia plug in gave normal results in both patients.

Using DBT2*, BBT2* and T1, 24 (17.4%), 21 (15.2%) and 76 (55%) patients had low values, suggesting cardiac iron overload. DBT2* and BBT2* were discordant in 7 patients with borderline iron overload (T1 was low in all). DBT2* and T1 were discordant in 52, always with a normal T2* and low T1, i.e. T1 mapping trebles the number of patients diagnosed with cardiac iron (Figs 2 and 3). Below 20ms, the relationship among DBT2*, BBT2* and T1 was strong (DBT2* vs BBT2* r2 = 0.95; DBT2* vs T1 r2 = 0.92; BBT2* vs T1 0.85, all p<0.001).

**Fig 4:**
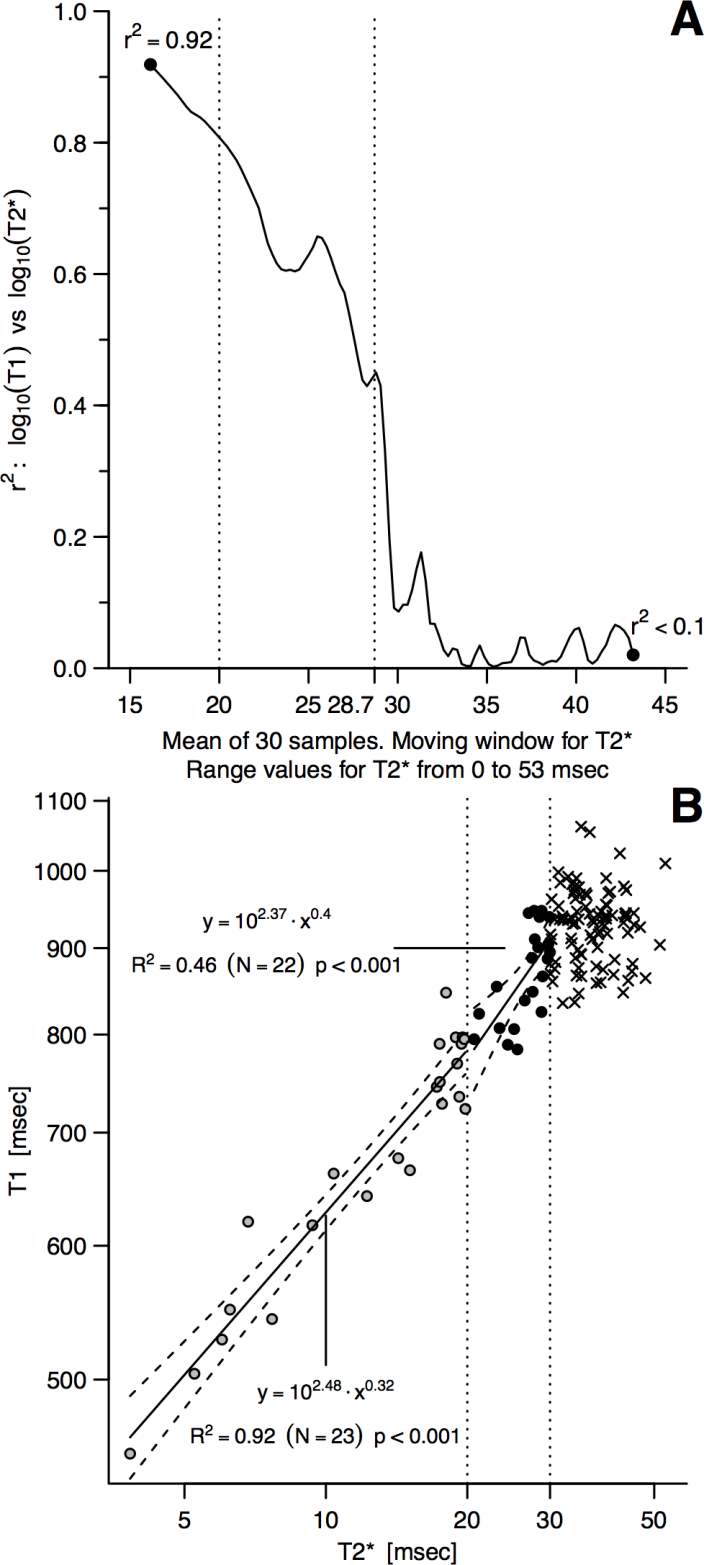
Myocardial T2*—T1 mapping relationship in Thalassemia major. (B): log-log linear regression lines for T2*—T1 (here Dark Blood T2*—MOLLI) in three different T2* intervals (T2* <20ms, T2* = 20-30ms and T2* >30ms)**–**for >30ms, the regression line is not drawn as the p value is 0.38 (weak relationship). (A): On the Y-axis are represented the r2 values of the relationship between T2* and T1 (both after log transformation). Data are obtained for a 30 samples moving window, with steps of 1 sample at a time, smoothed by a low-pass filter of order 3. Afterward, a new equispaced series was generated by linear interpolation. The point, corresponding to T2* = 28.7ms, was the local maximum found by setting the first derivative equal to 0, immediately before a large variation of the determination coefficient. A rounded value of T2* = 30ms has then been used to delimit a T2* range of 20-30ms, with suspect mild iron accumulation.

Using from now on only the DBT2*, T2* relationship with T1 was strong below 20ms and increasingly weak at higher T2*s. Fig 4 (panel B) shows three domains of linear regression: (a) strong relationship in the T2* = 0-20ms range (r2 = 0.92, p<0.001); (b) moderately strong relationship in the 20-30ms range (r2 = 0.48, p<0.001); (c) weak relationship above T2* = 30ms (r2 = 0.01, p = 0.193).

Fig 4 (panel B) shows how an interval of moderately strong T1-T2* linear regression was identified. A 30 samples moving window was computed over T2* with steps of 1 sample at a time. The local maximum, found by setting the first derivative equal to 0, immediately before the large variation of the determination coefficient, was at T2* = 28.7ms. In consideration of the intrinsic limitations of the methodology (constant decline and oscillation of the determination coefficient r2 by 10%, visual inspection of the curve), which make the precise definition of a cut-off impossible, for the purpose of this work such value has been rounded to T2* = 30ms, which is to be considered as part of an interval (T2* 20-30ms) and not as a sharp cut-off point.

## Discussion

This paper provides novel information on a different ability of T1 and T2* in detecting cardiac iron overload. The internationally accepted cut-point for iron load assessment by T2* of 20ms is known to be conservative–prioritising specificity over sensitivity. In our study T1 mapping trebles the number of patients diagnosed with cardiac iron overload, while T2* missed 2 out of every 3 patients with such an abnormality (Fig 3). The T1 vs T2* curve-fit relationship suggests that the actual T2* cut-point for normality (if T2* were a precise method at low iron levels) would be around 30ms. The value of 20ms was originally set because T2* becomes increasingly influenced by susceptibility artefact as iron levels fall resulting in increasingly poor reproducibility. The availability of a second test, T1 mapping, now further emphasises such a known limitation of T2* and brings new options into the clinical application of cardiac MRI.

T1 mapping standardization is progressing rapidly with two series phantom experiments, in 40 and 69 centres respectively, worldwide, [23,24] and could already be a useful complementary tool to T2* both for clinical and research purposes. The reported reproducibility of T1 (2.5–7 times T2*), squared for power calculations, would translate into 6.25 to 49 times more power in studies to detect cardiac iron change (i.e. more power for higher iron levels, at which the correlation T1/T2* is stronger). [25] Colour maps make iron instantly visible and add a useful confirmation step in the clinical diagnostic process. A reduced T1 is not entirely specific for iron, given that fat in the rare Fabry’s disease also causes reduced T1. Conversely, T2* appears highly specific, being altered only by the presence of gadolinium and (possibly, as suggested by a single case report), by cobalt/chromium. [26]

The clinical relevance of mild iron overload missed by T2* has not been clarified yet. In our cohort, a 24-month follow-up was available for only 9 patients with normal DBT2* and low T1. Although no statistical consideration is possible due to the small number, in those patients an increase in LV end diastolic volume was observed (from 78±18ml to 84±15ml), suggesting a possible cardiotoxicity of even mild amount of iron, although EDV changes may also be influenced by anaemia. Further work is needed, particularly in high risk groups like children starting chelation or around pregnancy. Another key-step is to determine whether T1 can detect changes in iron loading earlier, particularly during iron accumulation, because this would enable clinicians to modify chelation therapy earlier.

In conclusion, the potentially clinically relevant message we want to convey is that in the T2* 20-30ms range there might be iron load undetected by T2*. Thus, in such a scenario, T1 mapping could help, as it appears able to identify even small amounts of iron accumulating in the heart. Our observations, as well as their possible clinical implications, would need to be confirmed by future studies.

## Supporting information

S1 Fig Examples of T2* and T1 mapping images and analysis technique. Panel A: example of DB T2* sequences, followed by analysis results by CMR tools–Thalassemia tools plug in. Panel B: example of a T1 images, followed by in-line generated T1 map; on the map, a single region of interest was manually traced in the interventricular septum avoiding the endo- and epicardial contours. ROI: region of interest.
(TIFF)

S1 File Dataset used for analysis. Demographical, CMR and clinical data for the TM cohort.
(CSV)
