# Supplementary figures and images for: Role of T1 mapping as a complementary tool to T2* for non-invasive cardiac iron overload assessment
Source: PLoS One. 2018 Feb 21;13(2):e0192890. doi: 10.1371/journal.pone.0192890 (PMC5821344; doi:10.1371/journal.pone.0192890)

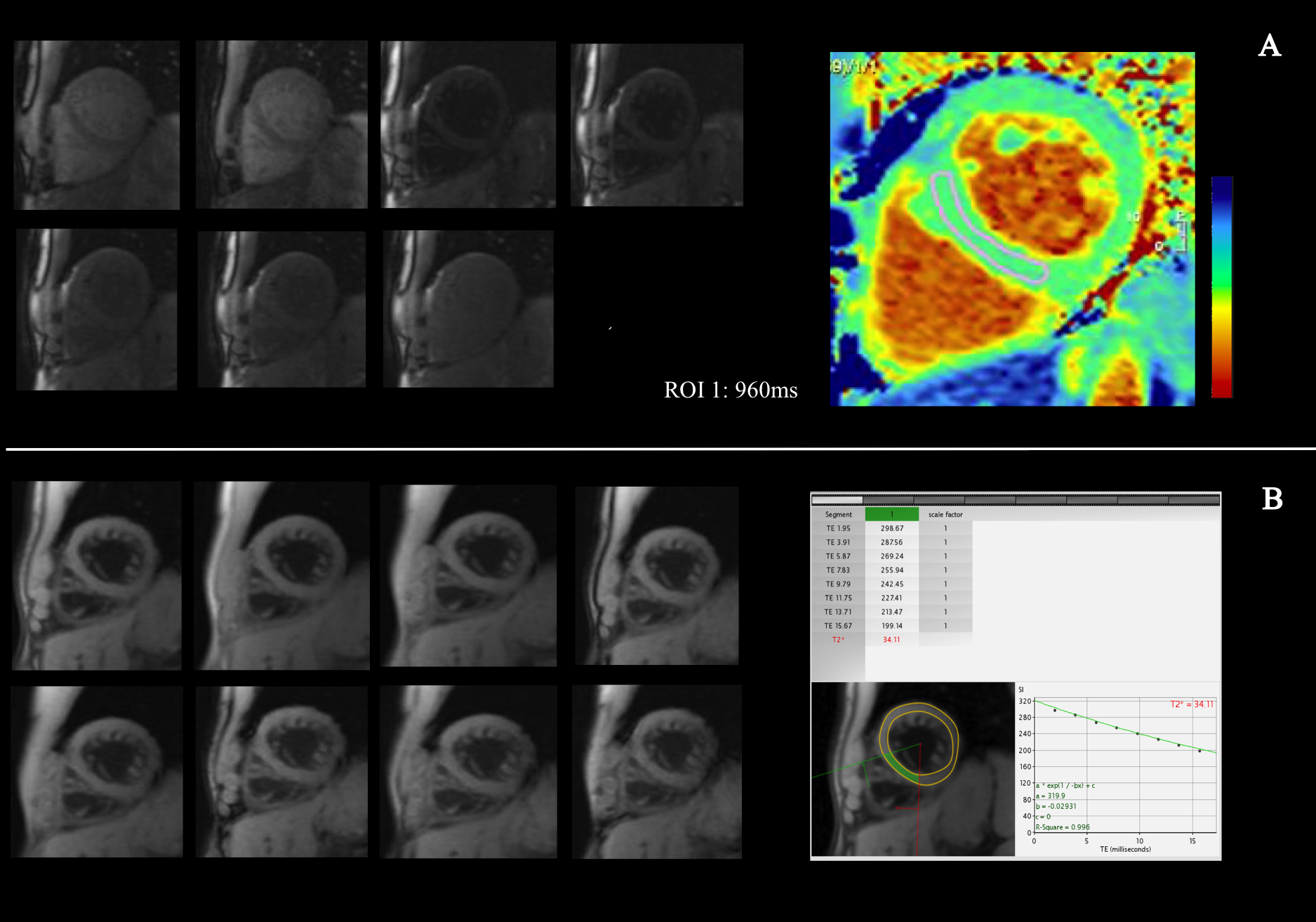

Supplement: S1 Fig — Panel A: example of DB T2* sequences, followed by analysis results by CMR tools–Thalassemia tools plug in. Panel B: example of a T1 images, followed by in-line generated T1 map; on the map, a single region of interest was manually traced in the interventricular septum avoiding the endo- and epicardial contours. ROI: region of interest. (TIFF) [file pone.0192890.s001.tiff]
